# The relationship between neutrophil–lymphocyte ratio and in-stent restenosis in superficial femoral artery

**DOI:** 10.1042/BSR20193448

**Published:** 2020-07-02

**Authors:** Yaobo Yang, Fangfang Ge, Jing Shen, Jianbo Song, Jiapei Xie, Jiangshuai Qu, Xinzu Mao, Zhaocheng Kuang, Xiang Wang, Yejun Wu, Shenghai Wang, Liang Xiao

**Affiliations:** 1 Department of Intervention,The fourth affiliated Hospital of China Medical University, Shenyang, Liaoning, China; 2 The First Affiliated Hospital of Dalian Medical University, Dalian, Liaoning, China; 3 Department of Computer Tomography Diagnosis, Yan’an People’s Hospital,Yan’an, Shaanxi, China

**Keywords:** diabetes mellitus, in-stent restenosis, Neutrophil-to-lymphocyte ratio, superficial femoral artery

## Abstract

The present study aimed to investigate the relationship between an increase in the pre- and post-operative neutrophil–lymphocyte ratio (NLR) and superficial femoral artery in-stent restenosis (ISR) rate. We recruited 199 patients that underwent superficial femoral artery stenting for lower extremity arteriosclerosis obliterans at our hospital from March 2015 to July 2018. Patients were divided into two groups according to the occurrence of ISR within 1 year (group 1, ISR and group 2, Non-ISR). The after NLR (NLR_after_) and NLR change ratio (NLR_ratio_) (*P*<0.001) were significantly higher in group 1. A NLR_after_ > 4.3 was associated with an odds ratio of 1.946 (95% CI [1.51–2.50]; *P*<0.001) for the presence of ISR. A NLR_ratio_ > 37.5% was associated with an odds ratio of 3.6 (95% CI [2.03–6.36]; *P*<0.001) for occurrence of ISR. A NLR_after_ level > 4.3 had 75% sensitivity and 76% specificity for the prediction of ISR, as identified by the ROC curve. A NLR_ratio_ level > 37.5% predicted ISR with 77% sensitivity and 60% specificity. Multivariate logistic regression analysis demonstrated that NLR_ratio_ was the strongest independent predictor of ISR (*P*<0.001). In conclusions, NLR_ratio_ could be used as a prognostic marker in superficial femoral artery stents.

## Introduction

Arteriosclerosis obliterans of the lower limbs is caused by the formation of atherosclerotic plaques of the lower limbs, which leads to arterial stenosis and occlusion, leading to chronic limb ischemia. Percutaneous superficial femoral interventions are the preferred treatment for superficial femoral artery disease, even though the occurrence of in-stent restenosis (ISR) continues to be an important complication [1,2]. The mechanism of stent restenosis is unclear. Neointimal proliferation underlies the pathophysiological development of ISR and is triggered by the pro-inflammatory molecules released due to endothelial damage, particularly during the thrombogenic and proliferative phases of ISR [3].

Recent studies have shown that inflammatory processes play an important role not only in the occurrence and development of atherosclerosis, but also in the occurrence of ISR [4,5]. The neutrophil–lymphocyte ratio (NLR) has been shown to be a marker of inflammation and closely related to increased cardiovascular mortality and morbidity [6,7]. Although the relationship between various inflammatory biomarkers, including NLR, and the occurrence of ISR has been investigated [8–10]. No studies have evaluated the effectiveness of the NLR value after (NLR_after_), NLR change(NLR_change_), defined as the NLR value before (NLR_before_) and after (NLR_after_) superficial femoral artery stenting (SFAS) intervention and NLR change ratio (NLR_ratio_), defined as the ratio of NLR_change_ to NLR_before_, in prediction of ISR in superficial femoral artery stents, which are at high risk for the occurrence of ISR. The present study aims to investigating the relationship between an increase in the pre- and post-operative NLR and superficial femoral artery ISR rate.

## Patients and methods

### Patient selection and research design

We recruited 199 patients that underwent SFAS for lower extremity arteriosclerosis obliterans at our hospital from March 2015 to July 2018. Inclusion criteria included the following: 1. Adult patients that underwent successful percutaneous transluminal stent implantation for superficial femoral artery lesions; 2. TASC-II classification of the femoral artery [1]: TASC-IIA, TASC-IIB, and TASC-IIC patients; 3. At least one arterial run-off below the knee, although stenosis lesions that were not limiting the flow may be included; 4. No evidence of residual inflow problems in the aortoiliac artery, although stenosis lesions that were not limiting the flow may be included. Exclusion criteria included the following: 1. Evidence of contraindications to anti-coagulation; 2. Active infection, chronic inflammatory disease, malignancy and chronic obstructive pulmonary disease; 3. evidence of hematological disease; 4. Presence of severe cardiac insufficiency (New York Heart Association grade III or IV), liver dysfunction (Child grade B or C) or renal insufficiency (creatinine clearance < 30 ml/min); and 5. No arterial run-off below the knee. All patients donated venous blood 3 days before and then after implantation. Routine hematological parameters including monocyte count, eosinophil count, basophil count, platelet count, platelet distribution width, neutrophil count, lymphocyte count, and levels of triglycerides, cholesterol, high-density lipoprotein, low-density lipoprotein, Bilirubin, Albumin, were measured by an auto-analyzer (Model XE2100; Sysmex Co, Kobe, Japan). Clinical and demographic data, and laboratory results were obtained from the hospital electronic medical records system for admitted patients. Hypertension was defined as blood pressure ≥140/90 mm/Hg or treatment with anti-hypertensive medications. Diabetes mellitus was defined as fasting glucose ≥126 mg/dl or treatment with oral anti-diabetic drugs or insulin. Smokers were defined as current cigarette users or patients who had quit smoking within 1 month of the procedure.

Before the procedure, patients received dual anti-platelet therapy consisting of aspirin (100 mg/d) plus clopidogrel (75 mg/d). The contralateral cross-over approach was used for all procedures. Interventional operations were performed through a 5F or 6F sheath (length 11–45 cm). Selective angiography was performed to localize lesions and measure the range of lesions by using 4F or 5F catheter. Balloon angioplasty was performed using the predilatation technique, and the diameter of balloon was determined by angiography. Self-expanding stents were implanted with a residual diameter stenosis >30% and/or flow-limiting dissection after balloon angioplasty in accordance with American College of Cardiology/American Heart Association guidelines [11]. Self-expanding stents had a diameter of 6 mm, and 5-mm diameter balloons were used for postdilatation within the stent. Percutaneous transluminal angioplasty success was defined as dilation of all arterial lesions with a residual stenosis of 20%. Stent technical success was defined as a residual stenosis of < 20% after stent placement. All interventional procedures were performed under local anesthesia. All patients received 5000 units of heparin during the procedures. After taking 100 mg of aspirin and clopidogrel at 75 mg for 12 months, clopidogrel was discontinued and administration of aspirin was sustained. Doppler ultrasonography, computed tomography angiography or digital subtraction angiography was performed every 3–6 months after stenting the superficial femoral artery. ISR was defined as not less than 50 percent stenosis in the treated lesion [12]. The study was approved by the local Ethics Committee of the Fourth Affiliated Hospital of China Medical University. All patients signed an informed consent.

### Statistical analysis

Data were analyzed using the SPSS version 21.0 software package. Continuous data were given as mean ± S.D. Discrete parameters were presented as percentages. The Kolmogorov–Smirnov test was used to evaluate a normal distribution. An independent-samples *t*-test was used to compare continuous variables between the two groups. The Χ^2^ test was used to compare categorical data. Entered factors included those with *P*<0.1 in univariate analysis. Logistic regression analysis was used to identify predictors of ISR. Receiver operating characteristic (ROC) curve analysis was used to determine the cutoff values of the NLR. A probability (*P*) value of <0.05 was considered statistically significant.

## Results

We enrolled 199 patients to the present study, all of whom were followed up for more than 1 year, of whom 80 (40%) were determined as having ISR. There were 155 (77.9%) men and 44 (22.1%) women in this group. Patients were divided into two groups according to the occurrence of ISR within 1 year (group 1, ISR; group 2, Non-ISR). There was no difference between groups for age and sex distribution. There were 108 (54.3%) smoking, 50 (25.1%) coronary heart disease, and 112 (56.3%) hypertension patients although none were statistically different when comparing the ISR and no-restenosis groups (*P*>0.05). In addition, the TASC II classification of the femoral artery was not statistically different between the ISR and no-restenosis groups (*P*>0.05). Χ^2^ test analysis the diabetes mellitus statistically different between both groups (*P*=0.028). The post-interventional monocyte count was significantly different (ISR = 0.76 ± 0.28 vs Non-ISR = 0.68 ± 0.26, *P*=0.031). Although the before NLR was not different between the groups (ISR = 2.87 ± 1.12 vs Non-ISR = 3.36 ± 1.99, *P*=0.051), the NLR_after_ was significantly different (ISR = 4.94 ± 1.35 vs Non-ISR = 3.65 ± 1.55, *P*<0.001) and the NLR_ratio_ was significantly higher in Non-ISR (ISR = 4.94 ± 1.35 vs Non-ISR = 3.65 ± 1.55, *P*<0.001) (Table 1).

**Table 1:** Clinical and hematological characteristics of the according to development of in-stent restenosis

	ISR, *n*=80 (%)	Non-ISR, *n*=119 (%)	*t*	*P* value
Male	59 (73.7%)	96 (80.7%)	Χ^2^ = 1.33	0.270
Age, Y	68.72 ± 11.37	70.59 ± 9.52	−1.256	0.211
Smoking	41 (51.2%)	67 (56.3%)	Χ^2^ = 0.492	0.562
Hypertension	42 (52.5%)	70 (58.8%)	Χ^2^ = 0.770	0.386
Diabetes mellitus	54 (67.5%)	61 (51.3%)	Χ^2^ = 5.170	0.028
CAD	22 (27.5%)	28 (23.5%)	Χ^2^ = 0.400	0.620
TASC II classification				
TASC A	3	4	0.980	
TASC B	40	59		
TASC C	37	56		
**Hematologic parameters**				
Total cholesterol, mmol/l	4.40 ± 1.28	4.23 ± 1.19	0.929	0.354
Triglyceride, μmmol/l	1.76 ± 1.37	1.88 ± 1.78	−0.520	0.604
HDL, mmol/l	1.04 ± 0.30	1.04 ± 0.31	0.043	0.966
LDL, mmol/l	2.65 ± 0.95	2.39 ± 0.95	1.926	0.055
Bilirubin, μmol/l	12.1 ± 0.32	11.9 ± 0.13	0.930	0.422
Albumin, g/l	34.0 ± 1.31	33.5± 2.12	0.870	0.541
**Hematologic parameters**				
Pre-interventional monocyte count	0.51 ± 0.17	0.55 ± 0.31	−1.101	0.272
Post-interventional monocyte count	0.76 ± 0.28	0.68 ± 0.26	2.179	0.031
Pre-interventional eosinophil count	0.18 ± 0.19	0.18 ± 0.15	0.288	0.774
Post-interventional eosinophil count	0.20 ± 0.17	0.21 ± 0.25	−0.033	0.974
Pre-interventional basophils count	0.04 ± 0.02	0.03 ± 0.02	1.173	0.242
Post-interventional basophils count	0.03 ± 0.01	0.04 ± 0.05	−0.842	0.401
Pre-interventional leukocyte count	7.95 ± 1.88	8.34 ± 3.11	−0.996	0.320
Post-interventional leukocyte count	8.62 ± 2.66	8.17 ± 2.86	1.114	0.267
Pre-interventional platelet count	229.62 ± 77.83	232.02 ± 91.70	−0.192	0.848
Post-interventional platelet count	210.50 ± 69.40	215.75 ± 85.39	−0.458	0.647
Post-interventional PDW,%	16.91 ± 0.88	16.61 ± 1.18	1.899	0.059
Post-interventional PDW,%	18.24 ± 0.65	17.47 ± 3.61	1.868	0.063
Pre-interventional neutrophil count	5.32 ± 1.61	5.23 ± 1.65	0.366	0.715
Post-interventional neutrophil count	9.22 ± 1.07	5.80 ± 2.04	13.719	<0.001
Pre-interventional lymphocyte count	2.00 ± 0.64	1.82 ± 0.66	1.888	0.061
Post-interventional lymphocyte count	1.95 ± 0.41	1.70 ± 0.52	3.643	<0.001
NLR_before_	2.87 ± 1.12	3.36 ± 1.99	−1.963	0.051
NLR_after_	4.94 ± 1.35	3.65 ± 1.55	6.071	<0.001
NLR_change_	2.07 ± 1.48	0.29 ± 1.86	7.136	<0.001
NLR_ratio_	0.93 ± 0.80	0.31 ± 0.63	6.099	<0.001

Abbreviations: CAD, coronary artery disease; HDL, high-density lipoprotein; LDL, low-density lipoprotein; NLR, neutrophil–lymphocyte ratio.

The predictors of ISR in the multivariate logistic regression analyses are presented in (Table 2). Because NLR_after_, NLR change(NLR_change)_ and NLR_ratio_ were inflammatory markers, they were not considered together in the multivariate model. Therefore, three multivariate models including NLR_after_ (NLR_after_, model 1), NLR_change_ (NLR_change_, model 2) and NLR_ratio_ (NLR_ratio_, model 3) were separately constructed. A NLR_after_ > 4.3 was associated with an odds ratio of 1.946 (95% CI [1.51–2.50]; *P*<0.001) for the presence of ISR. A NLR_change_ > 1.24 was associated with an odds ratio of 2.17 (95% CI [1.63–2.88]; *P*<0.001) for the presence of ISR; A NLR_ratio_ value >37.5% was associated with an odds ratio of 3.6 (95% CI [2.03–6.36]; *P*<0.001) for the occurrence of ISR. Among these, NLR_ratio_ was found to be significant independent predictor of ISR in the multivariate logistic regression analysis. Pearson’s correlation analysis showed that NLR_ratio_ was positively correlated with the onset time of ISR (*r* = 0.41; *P*<0.001).

**Table 2:** Multivariate analysis of predictors of ISR after superficial femoral artery stenting

Variables	Model 1 (NLR_after_)		Model 2 (NLR_change_)		Model 3 (NLR_ratio_)	
	OR (95%CI)	*P*-value	OR (95%CI)	*P*-value	OR (95%CI)	*P*-value
Post-interventional monocyte count	1.294 (0.38–4.35)	0.677	1.81 (0.52–6.33)	0.350	2.18 (0.67–7.05)	0.192
Pre-interventional lymphocyte count	2.272 (1.33–3.86)	0.003	0.80 (0.46–1.38)	0.437	0.78 (0.44–1.36)	0.389
LDL	1.275 (0.89–1.75)	0.182	1.43 (1.02–2.01)	0.037	1.36 (0.98–1.89)	0.059
Pre-interventional PDW	1.116 (0.79–1.55)	0.522	1.16 (0.82–1.66)	0.384	1.18 (0.84–1.64)	0.326
Post-interventional PDW	1.07(0.95–1.21)	0.251	1.05 (0.92–1.20)	0.453	1.05 (0.91–1.21)	0.463
Diabetes mellitus	0.59(0.30-1.19)	0.144	0.73 (0.36–1.47)	0.383	0.72 (0.37–1.42)	0.354
NLR_change_	—		2.17 (1.63–2.88)	<0.001	—	
NLR_ratio_	—		—		3.6 (2.03–6.36)	<0.001
NLR_after_	1.946 (1.51–2.50)	<0.001	—		—	

Abbreviations: LDL, low-density lipoprotein; NLR, neutrophil–lymphocyte ratio; PDW, platelet distribution width.

**Figure 1:**
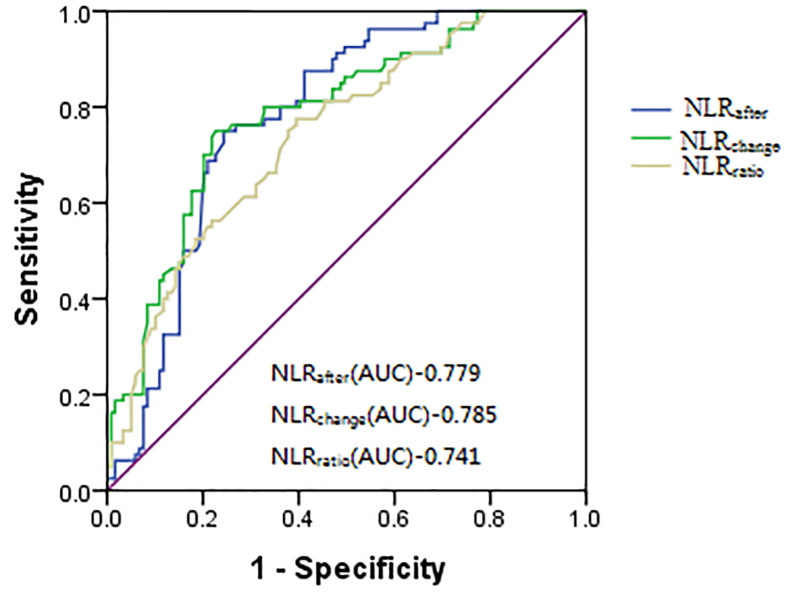
The ROC curves of NLR_after_, NLR_change_ and NLR_ratio_ were not significantly different, and the clinical diagnostic efficacy was almost the same NLR, Neutrophil–lymphocyte ratio.

A NLR_after_ level > 4.3 predicted ISR with 75% sensitivity and 76% specificity for the prediction of ISR, as identified by the ROC curve. The area under the ROC curve (AUC) was 0.779 (95% CI [0.71–0.84], *P*<0.001). A NLR_change_ level > 1.24 predicted ISR with 75% sensitivity and 77% specificity, AUC was 0.785 (95% CI [0.72–0.85], *P*<0.001). A NLR_ratio_ level > 37.5% had 77% sensitivity and 60% specificity. AUC was 0.741 (95% CI [0.67–0.81], *P*<0.001). The ROC curves of NLRafter, NLR_change_ and NLR_ratio_ were not significantly different, and the clinical diagnostic efficacy was almost the same. The ROC curve comparison of these three markers is shown in Figure 1.

## Discussion

Atherosclerosis is a progressive, complex and multifactorial disease. Inflammation plays an important role in all stages of the atherosclerosis development [13,14]. ISR has been attributed to neointimal hyperplasia in the early stage after the procedure [15]. Inflammatory cells may accelerate neointimal hyperplasia because of their release of growth and chemotactic factors [16] or production of enzymes (e.g. matrix metalloproteinases), which can degrade extracellular constituents and facilitate cell migration [17,18]. Neutrophils respond to different inflammatory stimuli resulting in release of various cytokines and cytotoxic/proteolytic enzymes that affect the vascular system by numerous mechanisms such as induction of damage to endothelial cells, induction of the coagulation system [19]. Lymphocyte count reflects a physiologic stress response to cortisol [20]. The NLR has been shown to be a marker of inflammation and closely related to increased cardiovascular mortality and morbidity [5,6]. The primary finding of our study is that the NLR_ratio_ level was a better independent predictor than NLR_after_ level for the occurrence of the ISR in patients who underwent superficial femoral artery stenting for lower extremity arteriosclerosis obliterans.

Our study observed that in patients presenting with restenosis within 12 months, the NLR increased after stent implantation. We find a positive correlation between the NLR_ratio_ and an occurrence of ISR (*r*=0.41; *P*<0.001). Patients with a NLR_ratio_ value > 37.5% had a 3.47-fold higher risk of ISR when compared with a NLR_ratio_ value < 37.5%. Patients with a NLR_after_ value > 4.3 had a 1.96-fold higher risk of ISR when compared with a NLR_after_ value < 4.3. Moreover, patients with a NLR_change_ > 1.24 had a 2.13-fold higher risk of ISR than did patients with a NLR_change_ value < 1.24. A NLR_ratio_ level > 37.5% had 77% sensitivity and 60% specificity for the prediction of ISR, A NLR_change_ level > 1.24 predicted ISR with 75% sensitivity and 77% specificity. The NLR_change_ in our study is higher than that reported in most other studies. For example, Balli et al. [21] reported the neutrophil–lymphocyte ratio for prediction of in-stent restenosis in coronary stents. A NLR_change_ level > 0.58 had 81.8% sensitivity and 93.5% specificity for the prediction of ISR. This discrepancy might be caused by the different lesion sites studied. The superficial femoral artery stent might be affected by the compression, pulling and torsion of the thigh muscle, resulting in continuous vascular damage, while the coronary arteries are less affected by the muscle. Chang et al. [10] reported that the NLR_before_ > 3.62 was independently and positively associated with a higher risk of early (within 1 year) ISR after stent implantation in patients with femoropopliteal chronic total occlusion. His results differ from ours in that we did not find a relationship between the NLR_before_ levels and the occurrence of ISR. The differences may be: first, we excluded patients with active infection, chronic inflammation, malignancy, and copd. Inflammation is considered to play a key role in the pathophysiological process for many chronic diseases. Second, they mainly studied the chronic complete occlusion of femoral popliteal artery, while we mainly studied the superficial femoral artery, and the patients did not have severe limb ischemia. In addition, the relationship between post-operative NLR and stent restenosis has not been analyzed.

Occurrence of ISR is a complex and multifactorial process [22]. Inflammation appears to be one of the many risk factors for stent restenosis [22,23]. The inflammatory process plays an important role not only in initiation and progression of atherosclerosis [14] but also in development of stent restenosis [24,25]. In general, the inflammation process contributes to stent restenosis by two distinct mechanisms, namely local vascular inflammation because of mechanical injury inflicted by stent implantation [24] and a pre-existing systemic inflammatory state before the procedure. [22,23]. The association between systemic inflammation and ISR has been reported in various studies conducted on different inflammatory markers. Of those, CRP was the most frequently studied biomarker owing to its accurately reflecting systemic inflammation [26] and being a strong predictor of cardiovascular outcomes [27]. Our research is mainly discussed because of mechanical damage caused by stent implants, namely, local vascular inflammation. Donners reported that [3] depending on endothelial damage during the PCI, increases in adhesive molecules and chemotactic factors are followed by the accumulation of inflammatory cells and pro-inflammatory molecules, such as interleukin-1 and 6, which mediate the development of neointimal proliferation. In our study, NLR_ratio_ was stronger independent predictors of short-term survival than other leukocytes. The predictive superiority of NLR may be due to 1 factor, NLR is a ratio of two different yet complementary immune pathways. Neutrophils are responsible for active ongoing nonspecific inflammation through secretion of many inflammatory mediators, like elastase [28], myeloperoxidase [29] and oxygen free radicals that can facilitate plaque disruption. An increase in neutrophil count predicts an increase in neointima and undesirable outcomes of stent restenosis. Lymphocytes, in contrast, represent the regulatory pathway of the immune system. CD4, the main subtype of total lymphocyte count, reflects a physiologic stress response to cortisol [20].The neutrophil–lymphocyte ratio therefore reflects both the neutrophil of inflammation and the relative lymphocyte of cortisol-induced stress response. In consequence, high NLR reflects two different immune pathways; hence, it is more predictive than either parameter alone.

In addition, we found that the proportion of diabetic patients in ISR group was significantly higher than that in non-ISR group, and the difference between ISR group and non-ISR group was significant. Diabetes mellitus has been shown to be an important risk factor for poor prognosis in patients with cardiovascular interventional therapy [30]. This is consistent with our observation that diabetes is an important factor for poor prognosis after superficial femoral artery stent implantation, but our findings suggest that diabetes is not an independent risk factor for ISR.

Our study has several limitations. This study was conducted on a retrospective basis and represented a single center experience. Second, the limitations of our study were the small sample, which we recognize might lead to differences in some of the reported observations.

In conclusion, NLR_ratio_ could be used as an inexpensive and easy-to-access method for assessment of inflammatory status and prognosis in patients with superficial femoral artery stents.

## Highlights

1. Determine the risk factors of restenosis in superficial femoral artery stents early in clinical treatment by simple and convenient examination methods.
2. Evaluate the studies that investigating NLR_ratio_ and to identify the prognostic and diagnostic value of NLR_ratio_ as an inexpensive and easy-to-access parameter after superficial femoral artery stents.
3. The new findings of this work have deepened the current understanding of in-stent restenosis in the superficial femoral artery and provide a theoretical basis for the prevention of ISR. As such the findings are valuable for future studies.

## Abbreviations

CAD: coronary artery disease
HDL: high-density lipoprotein
ISR: in-stent restenosis
LDL: low-density lipoprotein
NLR: neutrophil–lymphocyte ratio
PDW: platelet distribution width
